# Elevated Inflammation and Adhesion Molecule hsCRP, GDF-15 and VCAM-1 in Angina Patients with Non-obstructive Coronary Artery Disease

**DOI:** 10.21315/mjms2024.31.6.12

**Published:** 2024-12-31

**Authors:** Zulkefli Sanip, Aida Hanum Ghulam Rasool, Nurnajwa Pahimi, Nur Adilah Bokti, Zurkurnai Yusof, Mohd Sapawi Mohamed, W Yus Haniff W Isa

**Affiliations:** 1 Central Research Laboratory, School of Medical Sciences, Universiti Sains Malaysia, Kelantan, Malaysia; 2 Department of Pharmacology, School of Medical Sciences, Universiti Sains Malaysia, Kelantan, Malaysia; 3 Department of Internal Medicine, School of Medical Sciences, Universiti Sains Malaysia, Kelantan, Malaysia; 4 Cardiology Unit, Hospital Universiti Sains Malaysia, Kelantan, Malaysia; 5 Cardiology Unit, Hospital Raja Perempuan Zainab II, Kelantan, Malaysia

**Keywords:** coronary artery disease, C-reactive protein, growth differentiation factor 15, inflammation, non-obstructive CAD, vascular cell adhesion molecule-1

## Abstract

**Background:**

Non-obstructive coronary artery disease (NOCAD) is a condition in stable patients that experience angina despite not having significant coronary obstructive lesion. Knowledge on the role of certain biomarkers in patients with NOCAD is still limited. This study aimed to evaluate the roles of inflammation and adhesion molecules in the development of NOCAD. The correlations between the peripheral and coronary levels of the inflammatory biomarkers and adhesion molecules were also investigated.

**Methods:**

In this cross-sectional study, symptomatic angina patients scheduled for coronary angiograms were recruited and separated into obstructive coronary artery disease (OCAD) and NOCAD groups based on those angiograms. Peripheral and coronary blood samples were taken to measure inflammation biomarkers [high sensitivity C-reactive protein (hsCRP) and growth differentiation factor 15 (GDF-15)], and adhesion molecules [vascular cell adhesion molecule-1 (VCAM-1)]. Subjects without angina symptoms were recruited for the control group.

**Results:**

The hsCRP, GDF-15, and VCAM-1 levels were higher in the OCAD and NOCAD groups than in the control group. VCAM-1 levels successfully predicted the incidence of NOCAD [*p* = 0.010, area under the curve (AUC) = 0.716]. All biomarkers’ levels in the peripheral and coronary blood were correlated in OCAD and NOCAD patients (*p* < 0.001).

**Conclusion:**

Elevated levels of the hsCRP, GDF-15, and VCAM-1 were found with NOCAD, despite the absent of significant coronary obstruction. VCAM-1 successfully predicted NOCAD and may thus serve as an early NOCAD biomarker. Significant correlations of hsCRP, GDF-15, and VCAM-1 level in peripheral and coronary blood indicate that the peripheral levels of these biomarkers reflect the levels and changes that occur at the coronary level.

## Introduction

Non-obstructive coronary artery disease (NOCAD) is a condition that is increasingly being recognised in stable patients who present with evidence of coronary ischaemia but whose coronary angiogram does not show signs of obstructive coronary artery disease (OCAD). Such patients typically experience some symptoms, such as chest pain, abnormal stress testing, and other indicators of coronary ischaemia (1), and data has shown that they have an elevated risk of developing major adverse cardiovascular events such as death, nonfatal myocardium infarction and stroke, or experiencing rehospitalisation due to heart failure or angina (2). It has been previously reported that NOCAD patients have impaired peripheral microvascular reactivity, which suggests microvascular dysfunction (3).

There is substantial evidence supporting the involvement of inflammation in the progression of coronary artery disease (CAD) and high sensitivity C-reactive protein (hsCRP), an acute inflammatory biomarker that has been reported to be associated with CAD. In a study among patients who underwent coronary angiography, an independent association was observed between elevated levels of hsCRP and CAD, and hsCRP was found to be positively correlated with Gensini scores, indicating a relationship between hsCRP and the severity of CAD (4, 5).

Growth differentiation factor 15 (GDF-15) is a member of the transforming growth factor β superfamily (6), which is involved in regulating development, differentiation, and tissue repair in various organs (7). It is released from endothelial cells, macrophages, adipocytes, cardiomyocytes, and vascular smooth muscle cells, and as a stress-responsive cytokine, it can significantly increase in response to inflammation and tissue injury (8, 9). It has been reported to be involved in limiting macrophage activation and inflammation, in addition to its anti-apoptotic effects (7), and its upregulation has been recognised as an inflammatory biomarker with prognostic value in various conditions, especially cardiovascular disease (CVD). GDF-15 has been found to be a strong predictor of cardiovascular mortality in patients with CAD and coronary heart disease (10, 11). Higher levels of GDF-15 have been linked to a higher risk of mortality within a year in patients with ST segment elevation myocardial infarction (STEMI) and non-ST-elevation acute coronary syndrome (12, 13).

Vascular cell adhesion molecule-1 (VCAM-1) is a member of the cytokine-inducible immunoglobulin gene superfamily and is involved in leukocyte migration during inflammation. Inflammation-induced endothelium expresses VCAM-1, which mediates leukocyte binding and facilitates the migration of leukocytes into endothelial cells (14). For CVD, elevated levels of VCAM-1 have been reported to be associated with future cardiovascular mortality in CAD patients (15), and for patients with STEMI, VCAM-1 predicts post-STEMI heart failure and is associated with the development of new post-acute myocardial infarction heart failure symptoms (16). Another study reported that increased VCAM-1 levels in STEMI patients increased the risk for adverse clinical outcomes during the one-year follow-up period (17).

It thus appears that hsCRP, GDF-15, and VCAM-1, which acts as inflammatory biomarkers and adhesion molecules, play significant roles in various aspects of CVD (4, 5, 10, 11, 16, 17). The involvement of hsCRP has been reported in patients with angina symptoms but without significant coronary obstruction (NOCAD), but little is known about the involvement of GDF- 15, and more information regarding VCAM-1 is also needed. Knowledge of the correlation of these biomarkers between peripheral and coronary blood is similarly limited. Examining the associations and predictive value of these biomarkers in NOCAD patients may help to determine their possible roles in the mechanisms of NOCAD development and thus help to identify potential therapeutic targets. The current study therefore aims to: determine the levels of these biomarkers in people with NOCAD compared to those with OCAD and healthy controls; assess the ability of these biomarkers to predict NOCAD; and determine the correlations between the peripheral and coronary blood levels of these biomarkers in NOCAD.

## Methods

### Subjects

This observational cross-sectional study was conducted at the Hospital Universiti Sains Malaysia, Kelantan, Malaysia. The recruitment of participants started in March 2020 and continued until January 2022. The researchers invited symptomatic angina patients who were scheduled for coronary angiography at their invasive cardiology laboratory to participate. Nevertheless, patients with a history of cardiomyopathy, congestive heart failure, valvular heart disease, chronic kidney disease requiring haemodialysis, previous myocardial infarction, revascularisation procedures, or previous coronary angiography were excluded. A group of healthy individuals without any symptoms of angina or a history of ischaemic heart disease were also recruited as control subjects.

Patients’ weight and height were measured using a digital electronic weighing scale with an attached stadiometer (Seca, Hamburg, Germany). Systolic and diastolic blood pressures (SBP and DBP, respectively), were measured using a battery-operated automatic digital blood pressure monitor (HEM-7120, Omron, Kyoto, Japan). Left arm blood pressure measurements were taken twice in a sitting position at 5-minute intervals. Information regarding medical history was collected through interviews and also obtained from the medical report. The presence of cardiac risk factors among patients, such as diabetes mellitus, hypertension, hypercholesterolemia, family history of heart diseases, and smoking history was documented. Diabetes was defined as having high blood sugar levels [fasting blood glucose (FBG) > 7.0 mmol/L] that required dietary changes, medications or both. Hypertension was characterised by elevated SBP or DBP (> 140/90 mmHg) or requires antihypertensive medications. Hypercholesterolemia was identified as having a total cholesterol (TC) level above 5.2 mmol/L or being on lipid-lowering drugs. Current cigarette smoking referred to individuals who have actively smoked within the past year (18). Blood samples for glycaemic parameters [FBG and glycosylated haemoglobin (HbA1c)] and lipid profile analysis were promptly sent to an ISO-accredited clinical laboratory on the same day they were obtained.

### Coronary Angiography

The patients underwent standard preparations for coronary angiography in the invasive cardiology laboratory. An experienced cardiologist performed the procedure and obtained multiple views of the coronary angiography. Specifically, seven views were acquired for the left coronary arteries and three for the right. A quantitative computed tomography coronary angiography with an automated edge-detection system (Azurion, Philips, Amsterdam, Netherlands) was used to assess the occlusion percentage of the vessels, and any lesions present in the right coronary artery (RCA), left anterior descending artery (LAD), left circumflex artery (LCx), or their respective branches were documented. This study classified patients into two groups based on their luminal diameter reduction: those with a reduction of 50% or more due to stenosis in any of the main arteries (RCA, LAD, or LCx) were classified as obstructive, while patients without stenosis or with a luminal diameter reduction of less than 50% were classified as non-obstructive (19).

### Inflammation and Adhesion Molecule Biomarkers

Left main coronary blood samples were collected from patients through direct catheterisation before a contrasting agent was administered. All patients, including the healthy controls, also had blood samples obtained from the left forearm peripheral vein. The blood samples were allowed to coagulate at room temperature and then separated through centrifugation. The serum was transferred into small tubes, and the serum aliquots were stored at a temperature of −80°C until analysed. The concentration of hsCRP was quantified using commercially available DRG EIA kits (DRG International, NJ, US), and the concentrations of GDF-15 and VCAM-1 were determined using Elabscience ELISA kits (Elabscience, China). All measurements adhered to their respective manufacturers’ protocols. The absorbance was measured at a wavelength of 450 nm using Multiplate Varioskan Flash (Thermo Scientific, MA, US); a standard curve was then constructed and the sample concentrations calculated using SkanIt RE for Varioskan Flash software (Thermo Scientific, MA, US).

### Statistical Analysis

Data analysis was conducted using Statistical Package for the Social Sciences (SPSS) version 26 (IBM Corp., Armonk, NY, US). Continuous data were reported as mean (standard deviation, SD), while categorical data were presented as frequency (percentage). The comparison between the non-symptomatic control group and the OCAD and NOCAD groups was performed using one-way ANOVA for continuous variables, and the chi-square (χ^2^) test for categorical variables. Additionally, independent *t*-tests were performed for comparisons between two groups. Pearson correlation analysis was used to determine the correlation between parameters. Binary logistic regression analysis was conducted to assess the association and prediction of blood biomarkers on the likelihood of OCAD and NOCAD. A receiver operating curve was run to evaluate model fitness. A *p*-value <0.05 indicated statistical significance.

## Results

A total of 124 subjects participated in this study, of whom 43 were categorised as OCAD and 41 as NOCAD. Forty subjects with no symptoms of angina participated as the control group. Significant differences were found in DBP and lipid profiles between the groups. Cardiac risk factors, specifically diabetes, hypertension, hyperlipidaemia, family history of heart disease, and smoking, were more prevalent in the OCAD and NOCAD groups than the control group (Table 1).

One-way ANOVA post-hoc analysis revealed that the levels of peripheral hsCRP, GDF-15, and VCAM-1 were significantly higher in both the OCAD and NOCAD groups than the control group, but there was no difference between the OCAD and NOCAD groups (Table 2). The levels of hsCRP and VCAM-1 remained significantly higher in the OCAD and NOCAD groups than the control group, after controlling for potential covariates, consisting of age, gender, DBP, diabetes mellitus, hypertension, hyperlipidaemia, family history of CAD, smoking status, FBG, HbA1c, TC, triglyceride, and low density lipoprotein-cholesterol. For GDF-15, only the levels in the OCAD group were higher than the control group after controlling for these covariates (Table 3).

**Figure 1:**
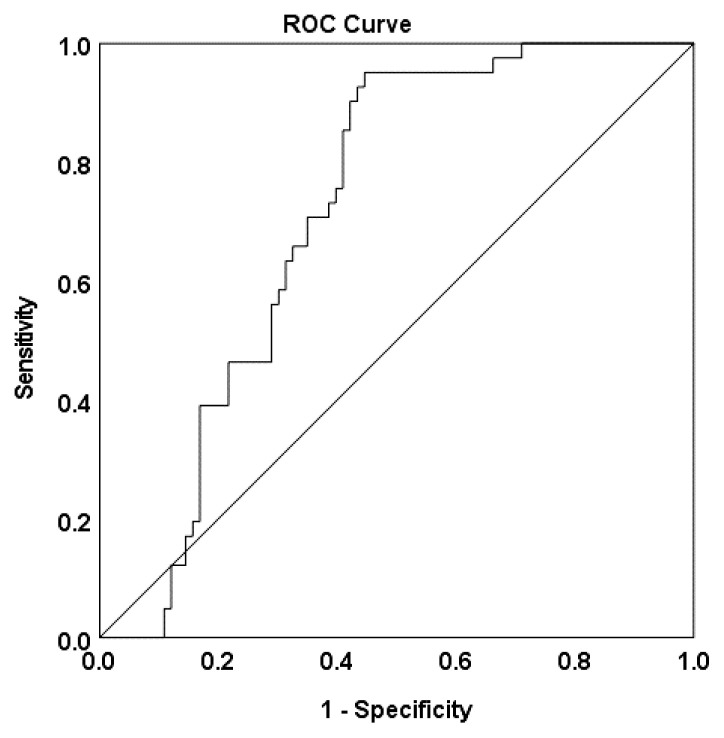
Receiver-operator curves showing accuracy for VCAM-1 to predict NOCAD in this study population (n = 124) Note: AUC = 0.716.

**Figure 2:**
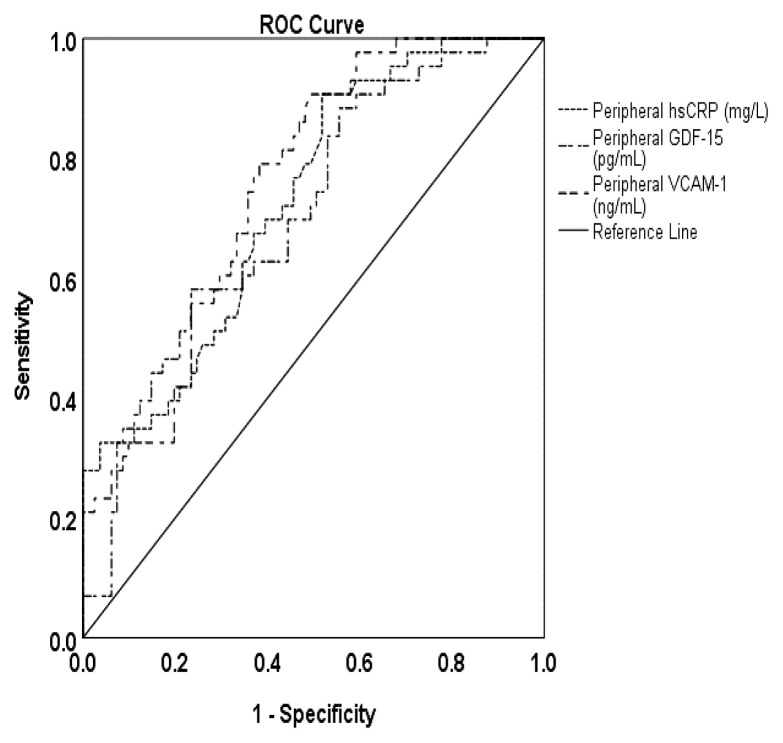
Receiver-operator curves showing accuracy for hsCRP, GDF-15, and VCAM-1 to predict OCAD in this study population (n = 124) Note: AUC = 0.728, 0.708, and 0.751 for hsCRP, GDF-15, and VCAM-1, respectively.

In the overall study population, the peripheral VCAM-1 level was found to be a significant predictor of NOCAD with an odds ratio (OR) of 1.004 (*p* < 0.010) (Table 4). The area under the curve (AUC) was determined to be 0.716 (Figure 1). This indicates that an elevated level of VCAM-1 significantly increases the risk of NOCAD by 1.004 times. Meanwhile, in the context of OCAD, it was observed that hsCRP (OR = 1.676, *p* = 0.013), GDF-15 (OR = 1.005, *p* = 0.012), and VCAM-1 (OR = 0.004, *p* = 0.016) levels were all significant predictors (Table 4), with corresponding AUC values of 0.728, 0.708, and 0.751 (Figure 2). This indicates that an increased level of hsCRP, GDF-15, and VCAM-1 significantly increases the risk of OCAD by 1.676, 1.005, and 1.004 times, respectively.

**Figure 3:**
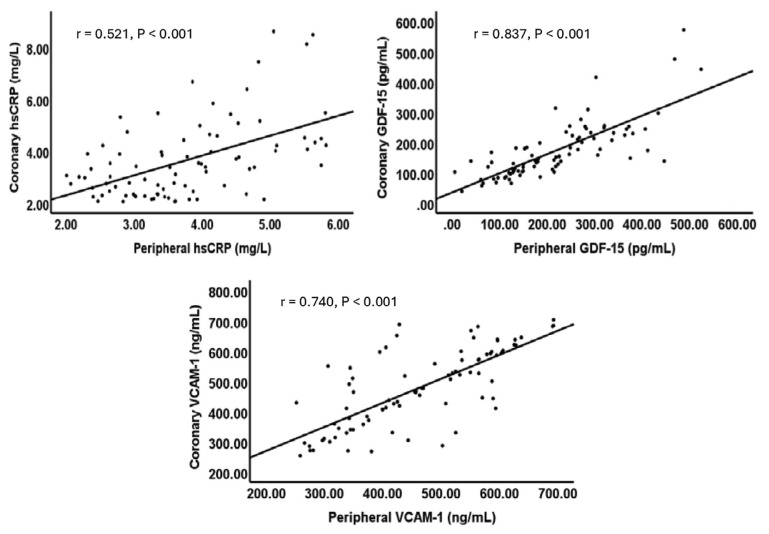
Correlations between peripheral and coronary inflammation biomarkers and adhesion molecule in symptomatic patients (n = 84)

Comparisons of coronary hsCRP, GDF-15, and VCAM-1 levels between the OCAD and NOCAD groups (n = 84) showed no significant differences (*p* = 0.403, *p* = 0.099, and *p* = 0.543, respectively). Furthermore, in symptomatic patients (OCAD and NOCAD groups), there were significant correlations between the peripheral and coronary levels of all three blood biomarkers (Figure 3).

## Discussion

In the current study, higher levels of the inflammatory biomarkers and adhesion molecules were observed in angina patients than in non-angina patients, irrespective of whether they had OCAD or NOCAD. In particular, this study found that VCAM-1 was a predictor for NOCAD, while all three parameters (hsCRP, GDF-15, and VCAM-1) were predictors for OCAD. Significant correlations were also found between peripheral and coronary levels of hsCRP, GDF-15, and VCAM-1.

Higher levels of hsCRP, GDF-15, and VCAM-1 in the OCAD and NOCAD groups than in the healthy control group, as found in the current study, are consistent with previous research that found elevated hsCRP, GDF-15, and VCAM-1 levels in OCAD patients compared to healthy controls (20–25). Higher hsCRP and VCAM-1 levels in NOCAD patients than in healthy controls have also been previously reported (20, 22, 24–26), but there is a lack of research data assessing the peripheral and coronary levels of GDF-15 in NOCAD patients. Notably, increased levels of hsCRP, GDF-15, and VCAM-1 indicate the involvement of inflammation and adhesion molecules in patients with ischaemic chest pain, regardless of the occurrence of stenosis.

In contrast, no significant differences were observed in the levels of hsCRP, GDF-15, and VCAM-1 between the OCAD and NOCAD groups, either peripherally or centrally; this is also consistent with previous research, which found similar levels of C-reactive protein and VCAM-1 in patients with OCAD and those with NOCAD or cardiac syndrome X (symptomatic with angiographically normal coronary arteries) in peripheral blood samples (24, 27, 28). However, there remains limited research comparing GDF-15 levels between OCAD and NOCAD groups.

In the current study, peripheral hsCRP, GDF-15, and VCAM-1 all significantly predicted the incidence of OCAD, but only VCAM-1 significantly predicted NOCAD. The AUCs for these biomarkers were all greater than 0.700, indicating acceptable discriminatory accuracy (29). Previous studies have reported the ability of hsCRP to predict both CAD and its severity (5, 20). In a study exploring a predictive model for coronary heart disease, hsCRP appeared as a key factor associated with CAD, whose addition to a predictive model increased its sensitivity and accuracy (30). GDF-15 was also found to be a good predictor of CAD, indicating its diagnostic value (21, 27, 31), and VCAM-1 was associated with higher incidence and greater severity of CAD (32, 33).

There is a close relationship between the biomarkers studied here, which are all part of the inflammatory process, and it is thus not surprising that they all increased in the OCAD group, and were associated with the presence of OCAD. During inflammation, pro-inflammatory cytokines, such as CRP, induce endothelial cell activation, which can manifest increased endothelial release of cell-surface adhesion molecules, including VCAM-1, which is in turn involved in leukocyte/monocyte migration from the circulating blood, through the endothelial cells, and into the vascular wall (14). Elevated levels of these molecules in the bloodstream lead to increased recruitment of monocytes to the cell membrane, where they aggregate as a colony. The accumulation of monocytes, alongside other inflammatory cytokines and fat cells that adhere to the cell surface, causes narrowing of the blood vessel, which directly affects the circulation of blood and may be associated with CVD conditions such as atherosclerosis and heart attack (34).

Meanwhile, GDF-15 is synthesised in activated macrophages in response to inflammation, vascular damage, pressure overload, and oxidative stress in human endothelial cells. Elevated levels of GDF-15 may be attributed to high levels of monokines, such as CRP; indeed, GDF-15 expression is induced by CRP through its control over p53 binding sites at the GDF-15 promoter. Although the precise biological functions of GDF-15 are not fully understood, studies have shown that it regulates inflammatory processes, cell death via apoptosis, and the formation of new blood vessels (35). Studies also demonstrate that GDF-15 plays a crucial role in advancing atherosclerotic lesions by modifying apoptotic cell death and interleukin-6-dependent inflammatory responses that result from vascular injury (36), implying that GDF-15 contributes to both the initiation and progression of atherosclerosis due to its central role as a pro-inflammatory cytokine (35).

It has been suggested that coronary microvascular dysfunction (CMD) is an underlying factor in NOCAD, in which patients experience angina without significant coronary artery obstruction (37), and previous studies have indeed reported a high prevalence of CMD in NOCAD patients (38, 39). In patients with cardiac syndrome X, peripheral hsCRP and VCAM-1 levels are inversely associated with coronary flow reserve (CFR), which is suggestive of CMD, while VCAM-1 has also been found to significantly predict CFR (40, 41). A study among female NOCAD patients demonstrated a significant association between GDF-15 and coronary flow velocity reserve, which is a parameter for accessing CMD (42). The association of these biomarkers with CMD highlights the possible role of inflammation in CMD, which presents with angina-like symptoms in NOCAD patients.

The current study found a positive association between VCAM-1 and the incidence of NOCAD, and every increment in VCAM-1 level significantly increases the likelihood of NOCAD. However, there is little similar evidence from previous studies. It has been suggested that, in patients with NOCAD, in which there is no significant stenosis or even normal coronary arteries, coronary microvascular endothelial dysfunction plays a significant role in the NOCAD pathologic mechanism (1). A previous study found that NOCAD group had impaired microvascular reactivity compared to a non-symptomatic group (3), and although the exact mechanism is still unconfirmed, prolonged inflammation may cause vascular dysfunction. The activation and adhesion of leukocytes to the vascular endothelium may be involved in the process and moderated by cellular adhesion molecules, including VCAM-1, which may play a part in microvascular angina (24). It can be postulated that higher levels of VCAM-1 result from the increased activation of endothelial cells in NOCAD patients, and a previous study discovered that endothelin-1, a powerful vascular endothelium vasoconstrictor, induces VCAM- 1 expression in small arteries (43). VCAM-1 has also been identified as the primary adhesion molecule in individuals with atherosclerosis, and is detectable on the surface of endothelial cells in the early stages of atherosclerosis development (44). Another study found that VCAM-1 expression is increased in atherosclerotic lesions, suggesting its critical role in atherosclerosis initiation (45). VCAM-1 may thus be a promising biomarker for early-stage CAD, especially in NOCAD patients who do not have significant stenosis.

The relationship between increased levels of VCAM-1 and the incidence of NOCAD in the current study also indicates the involvement of endothelial dysfunction, which is known to be an early precursor of atherosclerosis and is marked by the appearance of increased adhesion molecules, such as VCAM-1, in the endothelium (41). VCAM-1 also has appeared as a significant indicator of microvascular complications in type 2 diabetes, which would appear to establish its association with outspreading endothelial activation and dysfunction (46). VCAM-1 has already shown promising utility as a predictive biomarker for CVD, and the significant associations between VACM-1 and CVDs like hypertension, atherosclerosis, ischaemic heart disease, stroke, heart failure, arrhythmias, and atrial fibrillation have been discussed previously (47). These support the role of VCAM-1 as a biomarker for predicting the incidence, and continuation of CVD.

Similar to the peripheral blood levels, this study found no differences between the OCAD and NOCAD groups in their coronary blood levels of hsCRP, GDF-15, and VCAM-1. Furthermore, in all the symptomatic patients, significant linear correlations of the levels of these biomarkers between the peripheral and coronary blood were found. There are limited studies on correlations of the peripheral and coronary blood levels of GDF-15 and VCAM-1, but a study of stable and unstable angina patients identified significant linear correlations between the serum levels of hsCRP in the left forearm vein and coronary sinus for both groups (48). These findings indicate that measurements at peripheral vessels can reflect biomarker levels at the coronary vessels in angina, and peripheral blood collection has the advantage of being less invasive than coronary blood sampling.

The present study offered additional information to the current knowledge regarding the involvement of peripheral and central hsCRP, GDF-15, and VCAM-1 in NOCAD. Although there was no significant obstruction, NOCAD patients still suffered from angina. Increased levels of hsCRP, GDF-15, and VCAM-1 in both OCAD and NOCAD groups showed that all biomarkers play their roles regardless of stenosis status. Furthermore, the findings showed significant correlation of hsCRP, GDF-15, and VCAM-1 in peripheral and coronary blood, indicating the usefulness of peripheral measurement in assessing these parameter changes at the coronary blood vessel.

This current study may have limitations due to its cross-sectional design and convenience sampling method, including possible bias in patient selection and other confounding factors. Differences in age, comorbidities, and cardiac risk factors between the groups may affect the levels of hsCRP, GDF-15, and VCAM-1, although these factors were controlled for statistically in the covariate analysis, with the differences in the levels of the biomarkers persisting. Additionally, only a single measurement of the biomarkers was made, and thus changes in their levels over time, such as with the progression of OCAD or NOCAD could not be captured.

## Conclusion

Higher levels of the inflammation and adhesion molecules hsCRP, GDF-15, VCAM- 1 were found in angina patients with NOCAD than in non-symptomatic controls, but neither the peripheral nor coronary levels of these parameters differed between OCAD and NOCAD patients. Furthermore, hsCRP, GDF-15, and VCAM-1 were all significantly associated with the presence of OCAD, but only VCAM-1 was associated with NOCAD, which might indicate the involvement of endothelial dysfunction in the development of the angina symptoms seen in NOCAD patients and may thus be a valuable tool in identifying NOCAD. Significant correlations between peripheral and coronary blood in the levels of hsCRP, GDF-15, and VCAM-1 suggest the value of peripheral biomarkers in determining levels and changes at the lesion site.

## Figures and Tables

**Table 1:** Baseline characteristics of study participants (n = 124)

Parameter	Control (n = 40)	NOCAD (n = 41)	OCAD (n = 43)	*p-*value
Age (years)	49.85 (7.68)	51.07 (9.22)	54.30 (7.96)	0.044^a^
Gender, male/female, (n/n)	21/19	17/24	30/13	0.031^b^
BMI (kg/m^2^)	28.76 (4.37)	28.88 (4.66)	28.59 (4.33)	0.958
SBP (mmHg)	125.97 (12.59)	124.82 (17.75)	125.69 (17.85)	0.946
DBP (mmHg)	81.71 (8.54)	76.04 (10.19)	76.56 (8.01)	0.009^a^
FBG (mmol/L)	6.35 (3.83)	6.04 (2.33)	7.03 (3.21)	0.347
TC (mmol/L)	5.84 (1.24)	4.31 (1.14)	4.38 (1.43)	< 0.001^a^
TG (mmol/L)	1.99 (0.97)	1.17 (0.58)	1.21 (0.55)	< 0.001^a^
HDL-C (mmol/L)	1.33 (0.35)	1.22 (0.23)	1.21 (0.30)	0.118
LDL-C (mmol/L)	3.61 (1.21)	2.57 (1.07)	2.63 (1.29)	< 0.001^a^
Diabetes, n (%)	7 (17.5)	9 (22.0)	23 (53.5)	0.001^b^
Hypertension, n (%)	11 (28.2)	25 (61.0)	29 (67.4)	0.001^b^
Hyperlipidemia, n (%)	30 (75.0)	33 (79.1)	39 (90.7)	0.163^b^
Family history of heart diseases, n (%)	4 (10.3)	18 (43.9)	16 (37.2)	0.002^b^
Smoking status, n (%)	5 (12.8)	6 (14.6)	12 (27.9)	0.152^b^

Notes: Data presented as mean (SD) for continuous variables and frequency (percentage) for categorical variables.

a One-way ANOVA;

b Chi-square test;

BMI = body mass index; HDL-C = high density lipoprotein-cholesterol; LDL-C = low density lipoprotein-cholesterol; TG = triglyceride.

**Table 2:** Peripheral blood biomarkers measurements (n = 124)

Parameter	Control (n = 40)	NOCAD (n = 41)	OCAD (n = 43)	F-stat (df)	*p-*value
hsCRP (mg/L)	1.92 (0.63)	3.64 (0.77)	3.85 (1.22)	54.550 (2,121)	< 0.001*^a^,^c^
GDF-15 (pg/mL)	126.05 (75.86)	194.20 (110.33)	256.14 (167.01)	11.269 (2,121)	< 0.001*^b^,^c^
VCAM-1 (ng/mL)	226.55 (48.98)	452.98 (105.55)	466.41 (130.63)	71.234 (2,121)	< 0.001*^a^,^c^

Notes: Data presented as mean (SD).

* One-way ANOVA;

Post-hoc analysis with Bonferroni correction:

a NOCAD vs. control, *p* < 0.001;

b NOCAD vs. control, *p* < 0.01;

c OCAD vs. control, *p* < 0.001.

**Table 3:** Covariates analysis of peripheral blood biomarkers (n = 124)

Parameter	Control (n = 40)	NOCAD (n = 41)	OCAD (n = 43)	*p*-value
hsCRP (mg/L)	1.92 (1.49, 2.34)	3.70 (3.36, 4.03)	3.85 (3.52, 4.18)	< 0.001*^a^,^c^
GDF-15 (pg/mL)	132.23 (78.70, 185.76)	216.59 (174.12, 259.05)	236.54 (194.68, 278.41)	0.030*^b^
VCAM-1 (ng/mL)	243.47 (198.65, 288.94)	461.37 (425.81, 496.92)	446.32 (411.26, 481.37)	< 0.001*^a^,^c^

Notes: Data presented as adjusted mean [95% confidence interval (CI)].

* Analysis of covariance (ANCOVA).

Covariates: age, gender, DBP, diabetes mellitus, hypertension, hyperlipidaemia, family history of CAD, smoking, FBG, HbA1c, TC, triglyceride, low density lipoprotein-cholesterol. Pairwise comparison with Bonferroni correction:

a OCAD vs. control, *p* < 0.001;

b OCAD vs. control, *p* < 0.05;

c NOCAD vs. control, *p* < 0.001.

**Table 4:** Logistic regression analysis of peripheral blood biomarkers in predicting NOCAD and OCAD (n = 124)

Parameter	NOCAD (n = 41)			OCAD (n = 43)		

	OR	95% CI	*p*-value	OR	95% CI	*p*-value
hsCRP (mg/L)	1.354	0.931, 1.969	0.113	1.676	1.177, 2.517	0.013*
GDF-15 (pg/mL)	0.998	0.995, 1.001	0.257	1.005	1.001, 1.009	0.012*
VCAM-1 (ng/mL)	1.004	1.001, 1.007	0.010*	1.004	1.001, 1.007	0.016*

Notes:

* Indicates significant association using binary logistic regression. For NOCAD prediction, classification table = 65.3% and chi-square for model coefficient, *p* = 0.001; for OCAD prediction, classification table = 70.2% and chi-square for model coefficient, *p* < 0.001.

CI = confidence interval for OR.
